# A Methodology for Shielding-Gas Selection in Wire Arc Additive Manufacturing with Stainless Steel

**DOI:** 10.3390/ma17133328

**Published:** 2024-07-05

**Authors:** Felipe Ribeiro Teixeira, Vinicius Lemes Jorge, Fernando Matos Scotti, Erwan Siewert, Americo Scotti

**Affiliations:** 1 Center for Research and Development of Welding Processes, Federal University of Uberlandia, Uberlândia 38400-901, Brazil; teixeira.304@hotmail.com (F.R.T.); viniciuslemesj@hotmail.com (V.L.J.); 2 Linde GmbH, Linde Technologies, Department of Arc Technologies, 85716 Munich, Germany; fernando.scotti@linde.com (F.M.S.); erwan.siewert@linde.com (E.S.); 3 Department of Engineering Science, University West, 461 86 Trollhättan, Sweden

**Keywords:** arc-based AM, shielding gas, thin wall, austenitic stainless steel, metal transfer index, δ-ferrite

## Abstract

The main objective of this work was to propose and evaluate a methodology for shielding-gas selection in additive manufacturing assisted by wire arc additive manufacturing (WAAM) with an austenitic stainless steel as feedstock. To validate the proposed methodology, the impact of multi-component gases was valued using three different Ar-based blends recommended as shielding gas for GMA (gas metal arc) of the target material, using CMT (cold metal transfer) as the process version. This assessment considered features that potentially affect the building of the case study of thin walls, such as metal transfer regularity, deposition time, and geometrical and metallurgical characteristics. Different settings of wire-feed speeds were conceived to maintain a similar mean current (first constraint for comparison’s sake) among the three gas blends. This approach implied different mean wire-feed speeds and simultaneously forced a change in the deposition speed to maintain the same amount of material deposited per unit of length (second comparison constraint). The composition of the gases affects the operational performance of the shielding gases. It was concluded that by following this methodology, shielding-gas selection decision-making is possible based on the perceived characteristics of the different commercial blends.

## 1. Introduction

Conventional arc welding processes are widely used in wire arc additive manufacturing (WAAM) technology. Among those, GMA (gas metal arc) stands out due to its higher deposition rate. GMA requires a gas flow around the arc and the wire-electrode to protect the molten pool and the metal droplets in transfer from detrimental effects from the atmospheric air. For this reason, this gas is known as shielding gas. Shielding gases also stabilise the arc, control the operability and metal transfer, and influence the geometry, mechanical properties, and metallurgical characteristics of the deposit. The shielding gas must be selected according to the deposition material and application to fulfil these functions properly.

Regarding GMA in welding with austenitic stainless steels, Norrish [1] states that blends with argon with small additions of oxygen (1–2%) can be used when the metal transfer occurs in spray mode. Nobrega et al. [2] and Tasalloti et al. [3], in phase with Norrish’s recommendation, used blends of Ar + 2% O_2_ to weld successfully with austenitic stainless-steel wires (diameter 1 mm) with current around 200 A (conditions that may have led to spray transfer, based on the transfer maps presented by Scotti [4]). When short-circuiting metal transfer is employed, Norrish [1] warns that the appearance of the weld bead and the melting characteristics tend to be poor with Ar + O_2_ blends. In this case, a good alternative is to replace O_2_ with gases with greater heat exchange capacity. CO_2_, He, and H_2_ are highlighted for this purpose [5,6,7,8].

CO_2_ is the most popular constituent mixed with argon for austenitic stainless-steel short-circuiting welding. Ar-based blends with up to 5% CO_2_ can be used for welding austenitic stainless steels with good operational performance, but the resulting carbon level in the weld metal may rise above 0.04%; some authors [9,10] have reported that the increase in the CO_2_ content in shielding gas raises the carbon in the weld bead. This behaviour of high CO_2_ additions makes it unfeasible to use shielding gases with a high CO_2_ content, mainly for the base metal and wire class assigned with the character “L” in the acronym used in standards (maximum carbon content of 0.03%). In fact, several authors [11,12,13,14] employed low-carbon austenitic stainless-steel wires (AWS ER308LSi, AWS ER308L, and AWS ER316L) with blends of Ar with CO_2_ percentages only below 3%.

Helium is another constituent used in GMAW shielding gases for austenitic stainless steel. According to Mvola and Kah [15], Ar/CO_2_/He blends produce weld beads with a good appearance when using the short-circuiting transfer mode with stainless steels. Norrish [1] states that additions of helium in blends with Ar + CO_2_ flatten the beads and deepen the fusion of stainless-steel welds. In this direction, the results of Cai et al. [16] and Shackleton and Lucas [17] (yet using C–Mn steel wires) and Lozano et al. [18] (using an austenitic stainless-steel wire), show that additions of He increases weld bead penetration, depending on the contents of Ar and CO_2_ present in the blend. These findings concerning He addition in the shielding gas are important in welding operations, since faster travel speeds can be achieved without compromising the total penetration of the joint, resulting in reduced production time. These mentioned characteristics (less convexity and deeper penetration) are due to the high heat exchange capacity achieved with He. Furthermore, the higher ionisation potential of He can also contribute to these features if this gas property leads to higher heat input.

Still dealing with gases used for welding austenitic stainless steels, Norrish [1] states that there are proprietary ternary blends based on Ar/CO_2_/He with small additions of O_2_ and H_2_. According to Constanza et al. [19], adding H_2_ provides effects similar to He for these materials, even in small contents. Norrish [1] states that the addition of 1–2% H_2_ in gases with He content between 20–40% improves wettability (The beads are less convex). 

Extending the influence of the Ar-based shielding gases balanced with additives (O_2_, CO_2_, He, and H_2_) beyond wettability and penetration, their effects are also on arc stability, metal transfer regularity, microstructure, and mechanical properties. However, most publications covering these shielding-gas effects are unrelated to the stainless steel. Evaluating the short-circuiting transfer performance with a mild steel wire (AWS ER70S-6), Liskevych and Scotti [20] and Souza et al. [21] found that the higher the CO_2_ content in Ar-based blends, the more irregular the metal transfer. According to the first cited authors, the liquid meniscus abrupt rupture and consequent weld pool oscillation are more pronounced for increasing CO_2_ content, resulting in higher spatter generation rates. Besides that, these authors argue that the metal transfer regularity deterioration linked to higher percentages of CO_2_ in the shielding gas is due to incipient short-circuits and droplet deviation. Using a ferritic stainless-steel wire, Ferreira Filho and Ferraresi [22] verified that the addition of O_2_ or CO_2_ in Ar-based blends leads to an increase in the short-circuiting frequency and a reduction in the short-circuiting time, with the arcing time remaining practically constant. As mentioned by Jonsson et al. [23], the surface tension of liquid iron decreases as oxygen increases. Thus, with lesser resistance to droplet detachment, the drops are more easily released, reducing their size and increasing the short-circuiting frequencies. Evaluating the influence of helium content in a ternary shielding gas (Ar–CO_2_–He) mixture using a mild steel wire (AWS ER70S-6), Cai et al. [24] showed that a higher He content increases droplet size and decreases metal transfer frequency. Cai et al. [24] point out that the higher the He content, the lower the density of the shielding gas. Thus, the drag force of the plasma flow decreases, favouring larger droplets and lower transfer frequencies. 

Several studies have already evaluated the effect of shielding gases on the welding metallurgy of the materials, even though shielding gas in GMAW can be considered inert or slightly reactive. However, the gas type added to Ar and its content, the chemical composition of the deposited material, and/or the heat input intensity lead to different microstructures and mechanical properties. Using different ferritic stainless-steel wires, Ferreira Filho et al. [9] verified that the increase of CO_2_ in the shielding gas increased the carbon content in the weld metal, favouring martensite formation. Also evaluating the effect of various amounts of CO_2_ added to the argon-based shielding gas, but this time considering AWS ER70S-6 wire, Ebrahimnia et al. [25] showed that the volume fraction of acicular ferrite in the microstructure decreases and the volume fraction of Widmanstätten ferrite increases as the amount of carbon dioxide is augmented. They argued that the plasma turns warmer as the amount of carbon dioxide in the shielding gas increases, delivering higher heat to the plate (slower cooling rates), which leads to higher amounts of Widmanstätten ferrite. In response to the different ferrite morphologies, the hardness was reduced as the CO_2_ content increased, while the Charpy V-notch energy first increased and then remained constant. In another work, Açar et al. [26] studied the effect of three shielding gases (pure Ar, 3%H_2_ + Ar, and 7%H_2_ + Ar) on metallurgical aspects of joints manufactured with a martensitic stainless steel (base metal) using an AWS ER316L wire (austenitic stainless steel). The results showed a noticeable grain coarsening in the weld metal and heat-affected zone (HAZ) microstructures after adding H_2_ into the Ar gas during welding. Consequently, as verified by the tensile tests, the highest strength was obtained in the samples welded with pure argon. Gulenç et al. [27] also observed the same microstructural behaviour, evaluating different binary blends composed of Ar + H_2_ in joints welded with AWS ER304L wire (austenitic stainless steel). Gulenç et al. [27] justify that the higher thermal conductivity of the hydrogen causes more heat input into the welding zone, leading to a coarser microstructure.

It is important to understand that although there are similarities between welding and additive manufacturing, these processes have their particularities. For example, the cooling rates and the thermal history of the layers can be totally different, so not every shielding gas successfully used in welding is suitable for GMA, and vice versa. Although there are already studies that address the effect of different shielding gases on carbon steel [28], aluminium [29,30], and martensitic stainless-steel [31,32] parts built by GMA, an evaluation in terms of austenitic stainless steels has not been referenced in the literature yet, to the knowledge of this work’s authors. In addition, since the shielding gas is correlated with several GMA process variables, evaluating the effect of its change in composition is not trivial, as seen above for welding. To the author’s knowledge, there is no standardised or widely known procedure to compare the effect of shielding gases on the performance of GMA. 

Given this context, the first objective of this work is to propose and evaluate a systematic methodology for gas selection for GMA, emphasising a stainless-steel feedstock. Subsequently, to cover other gaps in the literature and validate the proposed methodology, the secondary objective is to evaluate and justify the impact of multi-component gases (with different physicochemical properties) used in Ar-based blends recommended as shielding gas for GMA of austenitic stainless steels. This impact will be assessed in terms of a wide range of features that potentially affect the built thin walls, such as metal transfer regularity, deposition time, and geometrical and metallurgical characteristics.

## 2. Materials and Methods

### 2.1. Methodology

A systematic methodology was conceived for this research. According to the material target of the study, a commercial austenitic stainless-steel gas metal arc wire used in welding was chosen for building up thin walls and evaluating the impact of different compositions of shielding gases on the alloy of GMA-manufactured builds. Three commercial Ar-based blends containing CO_2_, He, and H_2_ were used: Blend 1 (Ar + 2%CO_2_); Blend 2 (Ar + 2%H_2_ + 20%He + 500 ppm CO_2_); and Blend 3 (Ar + 1%CO_2_ + 1%H_2_). Three reasons justify the choice of these gases: a) they are commercial compositions and recommended by the manufacturer to weld stainless steels, becoming a valid comparison from a practical point of view; b) despite being Ar-based blends, the chemical additive contents are very different among them (more significant changes in physicochemical properties to better validate the shielding-gas selection proposal); c) they present contents within the ranges recommended by the literature considering the filler metal (austenitic stainless steel). Complementarily, a low-carbon “L” grade wire (resistant to sensitisation) was chosen as the only feedstock, to focus the comparisons only on the shielding gas. That is, the low-carbon grade wire will not disguise C pickup by the weld due to CO_2_ contents additively present in one of the shielding gases under study. As consumables (shielding gases and wire) were means, not ends, note that their brand names were omitted (they are not essential variables). 

Only one piece of arc welding equipment (gas metal arc, designed for short-circuiting metal transfer controlled by current and wire-feeding) operating with a single synergic line was employed for all three blends. To minimise even further the introduction of other dependent variables in the study, the same contact-tip work distance (CTWD) and number and length of layers were maintained between the three walls built with each shielding gas. As the same number of layers was maintained, the total wall height was expected to differ from gas to gas, but without hindrance to the study. 

Aiming at achieving the proposed objectives, this work was divided into two stages: preliminary and main experiments. In the preliminary experiments, a criterion was searched and, consequently, defined to allow a fair comparison among the gases. From the methodological point of view, an important experimental aspect had to be decided before running these preliminary experiments. By hypothesis, the gas additives can affect the shielding-gas characteristics (deposition rate, current, and voltage) even when using the same welding equipment and parameter settings. To understand the power source response in this regard, some exploratory tests were conducted in advance by maintaining the same parameter setting and replacing the shielding gases. When the same set parameters were kept with the three gas blends, apart from a variation in voltage (intrinsic due to the variation in the ionisation potential and the thermal conductivity of the gas), the actual mean wire-feed speed (WFS_m_) and the resulting mean current (I_m_) also changed (due to power-source control logic). Given these findings, some options were considered in the conceived methodology to establish a fair comparison between the different gases:

1. Keeping the same set values of wire-feed speed (WFS_set_) and deposition speed (DS) with the three shielding gases, but expecting different mean wire-feed speed (WFS_m_), mean current (I_m_), and, consequently, varied amounts of material mass deposited per unit of length (different WFS_m_/DS ratios);
2. Setting different WFS_set_ to reach the same WFS_m_ (actual WFS) for the three shielding gases (resulting in different I_m_), and, at the same time, adjusting the deposition speed (DS) to maintain the same amount of deposited material mass per length unit (same WFS_m_/DS ratio);
3. Setting different WFS_set_ to reach the same I_m_ (implying different WFS_m_) and, at the same time, changing disposition speed (DS) to maintain the same amount of deposited material mass per length unit (same WFS_m_/DS ratio) among the blends.

Varying the amount of material mass deposited per unit length when comparing different gas compositions is undesirable, because it would imply a proportional change in geometry and, therefore, in the heat flux through the wall (conductive heat transfer mode downwards the thin walls). Obviously, the different shielding gases must lead to some alteration in geometry and heat flow. However, minimising this by maintaining the nominal amount of material mass deposited per unit length (same WFS_m_/DS ratio) is a good practice to isolate the effect of each shielding gas. This aspect made option (a) less reasonable for the purpose of this work. Thinking now about the mean current (I_m_) effect on the weld bead formation in the GMA processes, welding current is the main factor in generating heat on the pool surface. In addition, Scotti and Ponomarev [33] explain that the arc pressure on the molten pool has a remarkable influence on the geometry of the weld bead. As this near-stagnation arc pressure directly correlates with the current intensity (since it is due mainly to the electromagnetic field of the arc), it is crucial to keep the same I_m_ to maintain the pressure as closely as possible when using different gases. It is said to be as close as possible, not the same, because the simple fact of using gases with higher density (i.e., Ar in contrast to He and H_2_) would already increase the stagnation pressure exerted by the plasma jet. Likewise, if the gases result in different arc column shapes (more or less conical), the pressure of the plasma jet must also change. Therefore, option (c) was chosen as the best parameter-setting alternative. That is, WFS was set to reach the same I_m_ (implying different WFS_m_) and, simultaneously, DS was adjusted to maintain the same amount of deposited material mass per length unit (same WFS_m_/DS ratio) when comparing the blends.

Finally, in the main experiments, the effects of the three Ar-based commercial shielding gases were assessed by taking as comparison quantities the metal transfer regularity, deposition time, and geometrical and metallurgical aspects of thin walls deposited by GMA, following the criterion raised in the first stage.

### 2.2. Experimental Setup

**Figure 1:**
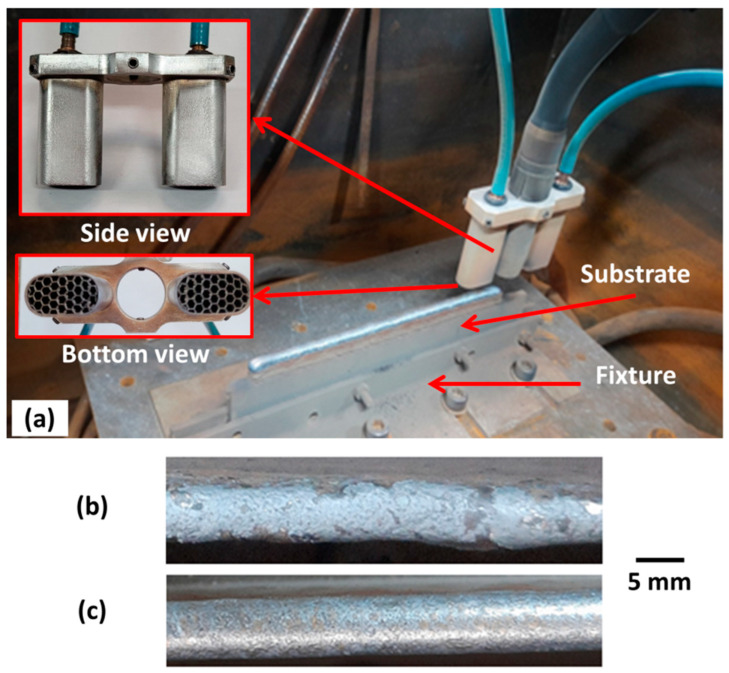
(**a**) Supplementary shielding-gas nozzles coupled to the welding torch, also emphasising the substrate and fixture during the wall building-up; the layer top surface appearance, (**b**) without and (**c**) with the use of the additional shielding.

The Fronius cold metal transfer (CMT) version of the GMA process was used as a power source and an AWS ER316LSi wire (1.2 mm diameter) was used as low-carbon grade austenitic stainless steel feedstock, shielded with the three gas blends under study (Blend 1, Ar + 2%CO_2_; Blend 2 Ar + 2%H_2_ + 20%He + 500 ppm CO_2_; and Blend 3 Ar + 1%CO_2_ + 1%H_2_). The synergic line CMT 928 (CrNi 19 9, ϕ = 1.2 mm, Ar + 2.5%CO_2_) was employed for all three blends, since no specific synergic lines for the blend with Ar + He or Ar + low hydrogen content were available at that time for the used equipment (Fronius CMT Advanced 400). Based on these settings, the process operated in short-circuit transfer mode. The contact-tip work distance (CTWD) was maintained at 12 mm during all depositions, being tracked with a reference gauge at each deposited layer. However, during the “preliminary experiments” stage, it was observed that the molten pool had an elongated profile (due to the low thermal conductivity of austenitic stainless steel and the slender wall over which the pools were formed). This elongated pool extended beyond the nozzle diameter. Thus, a supplementary shielding-gas (SSG) system was used, as illustrated in Figure 1a, to additionally protect the molten pool “tail” from atmospheric air. This same need has already been observed for aluminium [34]. Commercial high-purity Argon (Ar5.0) was employed as SSG at a flow rate of 15 L/min. As visualised in Figure 1b,c, the appearance of the layer top surface was considerably improved with this approach. 

The substrates were positioned in a fixture with the narrowest side facing up (working as a pre-wall), also seen in Figure 1a, aiming to keep the heat flux as constant as possible from the first layers. Flat carbon steel bars (220 mm × 50 mm × 6.4 mm) were used as the substrate, considering that several layers were planned to be deposited, allowing the discard of the first layers close to the substrate in the analysis. Similar stainless-steel bars were unavailable during the experiments, but this material choice did not influence the results for the given reasons.

A total of 30 layers were deposited over each substrate. The bidirectional deposition strategy was used, that is, the deposition of a new layer always occurred in a reverse direction to the previous one. No cooling approach (active or passive) was used during depositions, but the interlayer temperature was fixed at 50 °C (natural cooling). Therefore, heat accumulation along the wall-building direction was minimised by preventing higher temperatures before resuming a new layer. Consequently, the formation of deleterious phases was also prevented, such as the sigma phase, which requires long holding times within a temperature range between 600 and 900 °C [35], or sensitisation (carbide precipitation at temperatures ranging from about 450 to 850 °C, being the peak of nucleation rate between 600 and 750 ℃ in most austenitic SSs, according to Yin et al. [36]). The same method detailed in Jorge et al. [37] to monitor the interlayer temperature (named the upper pyrometer) was used, with a set emissivity of 0.63 (this work had a different surface emissivity than that of Jorge at al. [37]), making the experiments more repeatable and the results more comparable. Basically, a pyrometer measured the temperature at a given distance from the arc on the top surface before resuming a subsequent layer deposition. Due to the SSG nozzles, a space of 40 mm was used between the measuring point and the centreline of the wire. 

An A/D (analogic to digital conversion) board, operating for 8 s at an acquisition rate of 5 kHz and 14 bits (signal resolution), was employed to monitor the electrical signals (current and voltage) and wire-feed speed. The mean current and voltage and the average values of wire-feed speed and instantaneous power (the point-to-point product between current and voltage divided by the number of points) were determined for each layer. The arc energy per unit of length was determined by the ratio of the averaged instantaneous powers and the deposition speed used, as proposed by Scotti et al. [38].

### 2.3. Proposed Assessment Parameters

According to the conceived methodology for comparing the effects of the Ar-based shielding blends on the short-circuiting GMA deposition of austenitic stainless-steel thin walls, the impact of three shielding gases was assessed by some resulting arc- and wall-related characteristics, such as the metal transfer regularity, deposition time, and geometrical and metallurgical aspects of thin walls deposited by GMA. Since there are no standard methods for these assessments either, those assessment factors proposed and used in this study are described and justified in detail below. The authors of this study believe that such care with the assessment procedures is vital for reliable comparisons. This approach is not only to accomplish the global objective of this work (a systematic methodology for shielding-gas selection) but also the secondary objective (to understand the impact of the gas additives of the Ar-based blends on the resultant features of the wall).

#### 2.3.1. Effect of the Shielding Gas on the Metal Transfer Regularity in Short-Circuiting Mode

Knowing the challenge of evaluating the metal transfer regularity, qualitative and quantitative methods were employed during preliminary and main experiments, as follows:

- Qualitative analyses were conducted by visually observing the oscillograms (current × time and voltage × time graphs) and cyclograms (voltage × current graphs) resulting from the layer deposition with the three shielding gases. The regularity of the oscillograms can be ascertained when there is maintenance and periodicity of the current and voltage curves as a function of time. The regularity in cyclograms, in turn, will be more significant when the superposition of voltage × current signals is more concentrated;
- Quantitative analyses were carried out by using a metal transfer regularity index, hereafter referred to as IV_sc_ index. For that, mean values and standard deviations of short-circuiting frequency (F_sc_), arcing time (t_arc_), and short-circuiting time (t_sc_) were calculated (Equation (1)) using voltage traces of 25 layers of each wall, aided by a computer program developed and registered by Vilarinho e Araújo [39]. As described by Jorge et al. [40], the IV_sc_ index is closely linked to the consistency of arcing and short-circuiting durations. In other words, the lower the IV_sc_ value, the more regular the metal transfer.
  (1) $${IV}_{sc}=\frac{{\sigma t}_{sc}}{t_{sc}}+\frac{{\sigma t}_{arc}}{t_{arc}}$$
  where σt_sc_ and σt_arc_ represent the standard deviation of the monitored short-circuiting time (t_sc_) and arcing time (t_arc_), respectively, averaged along the sample duration.

In addition, one can still say that a longer t_arc_ in relation to the cycle duration means a proportionally greater arcing time (resulting in more energy being generated in the anodic region per cycle), while a longer t_sc_ indicates a more extended period of Joule heating effect along the wire delivered to the pool. Consequently, the t_arc_/t_sc_ ratio can roughly represent the heating efficiency of the arc in short-circuiting transfer; heat generated at the anodic region provides more heat to the plate than if delivered from the molten wire, regardless of the consumed electrical power. Therefore, the increase in this ratio for the same arc energy suggests higher heat input.

#### 2.3.2. Effect of the Shielding Gas on the Geometrical Features

Four geometrical features were quantified in this proposal, i.e., external width, effective width, surface waviness, and layer height. Concerning the layer height (LH), the total height of each wall was measured at five distinct positions of the layer extension, using an analogue calliper (0.05 mm resolution). These values were divided by the number of layers deposited so that the layer height averages and their respective standard deviations could be quantified. The same methodology first described by Jorge et al. [34] and Teixeira et al. [41] was used to measure the other three geometrical features. For this, the walls were scanned by a 3D Handy Scan (0.1 mm resolution) and the files were treated using a dedicated software. The functional area of the central region evaluated via the 3D scanner was 180 mm × 50 mm. For each wall, the software extracted the point clouds from 360 sections. Then, the code split each cross-section into two profiles, one for each side of the wall (left and right) and divided each side profile into small sample lengths (λ_c_) to minimise the influence of possible outliers. The λ_c_ value is the average layer height (LH) quantified for each wall. The software calculated the maximum and minimum values from the difference between the profiles for each λ_c_. After that, the code provided the average values per section for each side of the walls. The averages of the maximum and minimum values from both sides of the wall were summed to determine the external wall width (WW_ext_) and the effective wall width (WW_eff_), respectively, always considering the same profile. The surface waviness (SW) of the wall, in turn, was the average of the differences between the maximum and minimum values from both sides of the wall, considering the same profile, obviously. Finally, the averages and the respective standard deviations of WW_ext_, WW_eff_ and SW were obtained from each of the 360 sections.

#### 2.3.3. Effect of the Shielding Gas on Total Deposition Time

An estimation of the total deposition time T_Dt_ was proposed to assess the production efficiency promoted by each shielding gas. According to this proposal, T_Dt_ is quantified by Equation (2).

(2) $$T_{Dt}=\sum_{k=1}^{NL}D_{t}xI_{t}$$

where NL is the number of deposited layers, D_t_ is the deposition time of each layer, and I_t_ is the idle time to reach a given interlayer temperature between layers of the given layer. 

As Equation (2) can be manipulated numerically without experiments, different dummy idle times between layers (I_t_) were assessed to verify the weight share of this factor in T_Dt_. D_t_, in turn, was calculated by dividing each layer length by DS (deposition speed) employed with each gas, while NL was determined by dividing the target wall height by the average layer height achieved with each blend. However, as previously mentioned, the deposition speed (DS) was not the same among the depositions with the three shielding blends, aiming at maintaining the WFS_m_/DS ratio with each shielding gas. 

As will be shown, the external and effective wall widths were not precisely the same between the evaluated conditions. Thus, for comparison purposes, it became necessary to normalise the wall width between the three blends and consider parameters capable of achieving the same target width during the calculations with Equation (2). For this reason, the deposition speed (DS) was again adjusted for each gas using a normalisation factor, aiming to reach the same apparent effective width. This factor is taken from the simplification that the width and height of the layer would vary proportionally with the change in the layer volume, which, in turn, has a direct relationship with the WFS_m_/DS ratio. Therefore, if the apparent effective width target is 10% smaller than the effective wall width achieved with a given gas, an increase of 10% is applied in DS (reducing the pool volume and implying a shorter D_t_). The same reasoning was applied to the layer heights, resulting in different NL reaching a target wall height with each gas. That way, an effective wall width of 5 mm and a wall height of 100 mm were arbitrarily set as targets for the calculations. Furthermore, aiming to estimate the influence of the shielding gas on the total deposition time in terms of different situations, the T_Dt_ calculations arbitrarily considered an idle times (I_t_) range between 0.3 and 28 min (always assuming the same I_t_ between gases—in practice, these values may be different between them) and different wall lengths (200, 1000, and 5000 mm), which implies variations in deposition times (D_t_).

#### 2.3.4. Effect of the Shielding Gas on the Microstructure and Hardness

**Figure 2:**
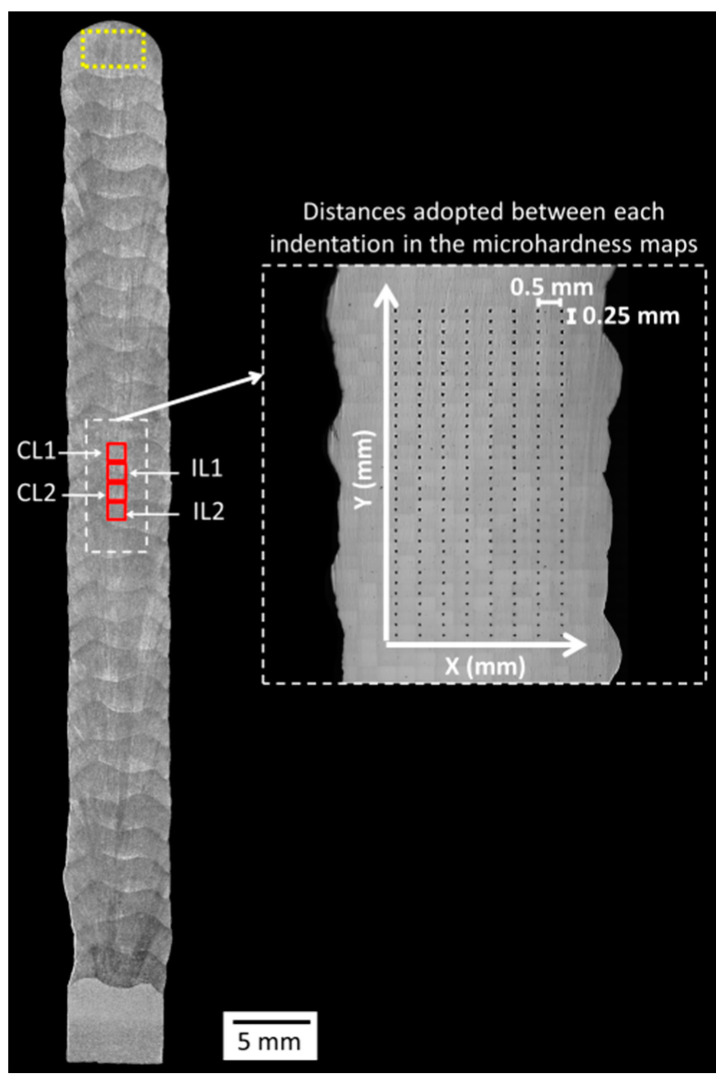
Positions of micrographic analyses (red solid-line squares), δ-ferrite quantification (small white dashed-line rectangle), and hardness map (large white dashed-line rectangle); the microhardness measurement region is magnified to show the distances adopted between each indentation.

A cross-sectional sample was taken from the centre of each wall length. After sand grinding and polishing, microhardness maps were performed at the centre of the cross-sections, the position spotlighted as a white dashed-line rectangle in Figure 2. For each map, 256 indentations were equidistantly engraved in an 8.0 mm × 3.5 mm area. The tests employed a load of 500 gf (4903.3 mN) and a time of 15 s. After the microhardness tests, the same samples were etched with aqua regia (HCl + HNO_3_), and four optical microscopy micrographs were obtained from each sample at the positions also indicated in Figure 2 by red solid-line squares. The micrographs were taken aiming at the centre of layers (CL1 and CL2) and interlayer regions (IL1 and IL2). Finally, a portable Feritscope device was used to estimate the δ-ferrite content along the centre of the cross-sections (same region delimited by the white dashed-line rectangle in Figure 2). A total of 30 measurements were performed in this region. Furthermore, to estimate the δ-ferrite content in the as-solidified condition (without suffering reheat by successive layers), 10 other measurements were conducted on the uppermost deposited layer (yellow dashed-line rectangle in Figure 2).

## 3. Results and Discussion

### 3.1. From the Preliminary Experiments

Recall that the preliminary series of experiments were conducted to find a combination of set wire-feed speed (WFS_set_) and deposition speed (DS) for all gases under assessment. To maintain the heat flow between the wall and the substrate as uniformly as possible, an attempt was made to maintain the wall width as close as possible to the substrate thickness (6.4 mm). In another column of the methodology presented in Section 2.1 to compare the blends, it was demonstrated that WFS should be set to reach the same mean current I_m_ (even though implying different WFS_m_) and, simultaneously, DS adjusted to maintain the same amount of deposited material mass per length unit (same WFS_m_/DS ratio). 

As the Blend 1 (Ar + 2%CO_2_) composition is the closest to that recommended for the CMT synergic line in use (see Section 2.1), this blend was defined as a reference. For tunning the parametrisation, the arc length (AL) and dynamic correction (DC) parameters of the CMT process were trimmed to reach the lowest possible spatter formation and a stable metal transfer. Eventually, a setting of WFS_set_ = 4.9 m/min, DS = 35 cm/min, AL = −3.0, and DC = −4 achieved the target wall width, with minor spattering. This combination resulted in an IV_sc_ index of 0.05 (indicating a very regular short-circuiting metal transfer), considered suitable for preliminary experiment purposes. The electrical parameters averaged between the layers provided I_m_ = 112.7 ± 0.8 A and WFS_m_/DS ratio = 10.9 ± 0.2 (a dimensionless quantity), considering that the average measured WFS was shown to actually be 3.8 m/min (WFS_set_ > WFS). Then, the two target reference values for comparison purposes were defined as I_m_ = 113 A and WFS_m_/DS ratio = 10.9.

**Figure 3:**
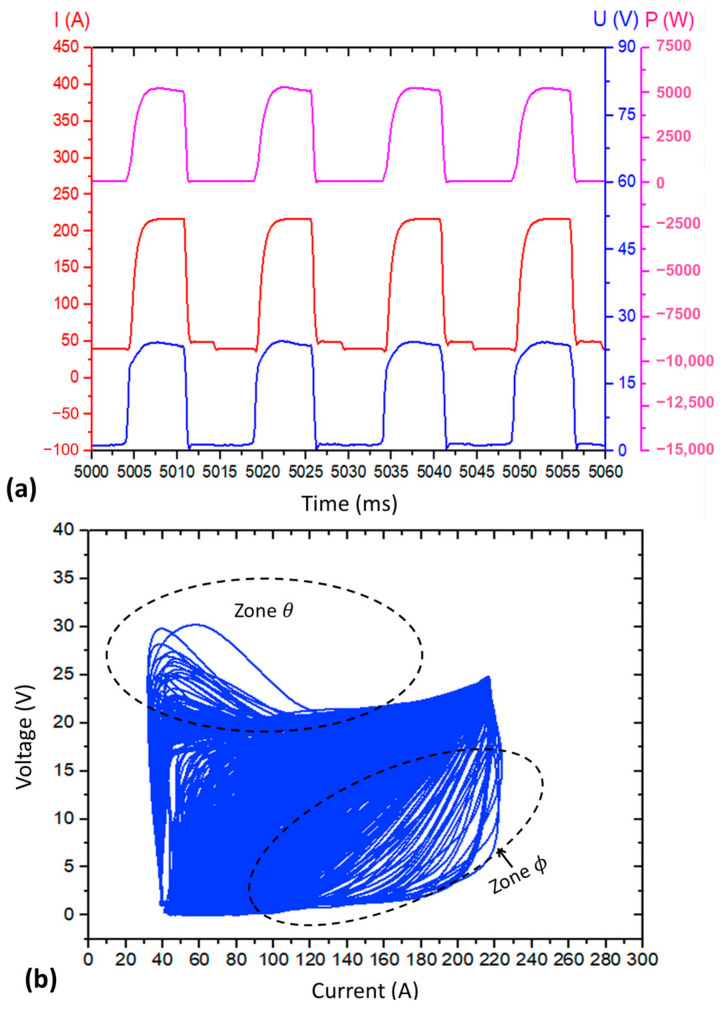
Wall deposition with Blend 1 (Ar + 2%CO_2_), WFS_set_= 4.9 m/min; DS_set_ = 35 cm/min, CA = −30 and CD = −4): (**a**) Current (I), voltage (U), and power (P) oscillograms; (**b**) cyclogram of voltage × current.

Applying the defined assessment parameters in Section 2.3.1, Figure 3 illustrates the current, voltage, power oscillograms, and the cyclogram obtained for Blend 1 (Ar + 2%CO_2_) from this preliminary experimental stage. Despite the high metal transfer regularity quantified by the IV_sc_ of 0.05 (the lower, the better) and verified by visualisation of the oscillogram of Figure 3a (regular and periodic behaviour of current and voltage signals), the cyclogram shown in Figure 3b presents high dispersion between the voltage × current traces. These traces are formed by the different arcing and short-circuiting cycles, mainly within the two highlighted zones (θ and ϕ) outlined in the side (b) of the figure.

**Figure 4:**
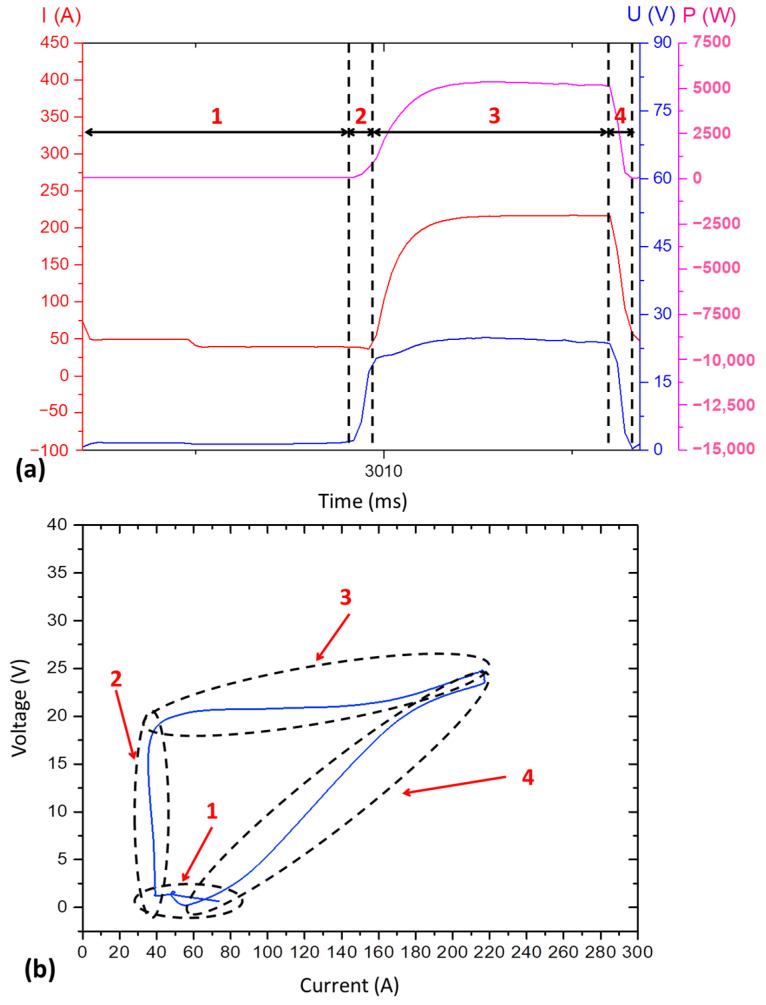
(**a**) Oscillograms of current (I), voltage (U), and power (P) for a typical CMT cycle; (**b**) respective voltage × current cyclogram, with the feature regions (1 to 4) that characterise the U and I traces.

However, to assess the cause of the above-mentioned zones θ and ϕ, it is necessary to understand each region that makes up a cyclogram. For that, Figure 4a shows an example of a single CMT cycle sampled from the same data used to build Figure 3a. Figure 4b, in turn, reproduces the cyclogram of this single cycle. The different regions numerically highlighted in the oscillogram of Figure 4a can be correlated with those represented in the cyclogram of Figure 4b. As seen, four regions are underlined as follows: region 1 corresponds to the beginning of the short-circuiting time, with voltage tending to zero and low current; region 2 corresponds to the beginning of the arcing time, where the arc length starts to increase (characterised by the voltage increase), but still at a low current; region 3 constitutes most of the arcing time and is established when the current starts to increase, but already at a high voltage (long arc length); region 4 is the one that precedes a new short-circuit, being characterised by the reduction of voltage and current.

**Figure 5:**
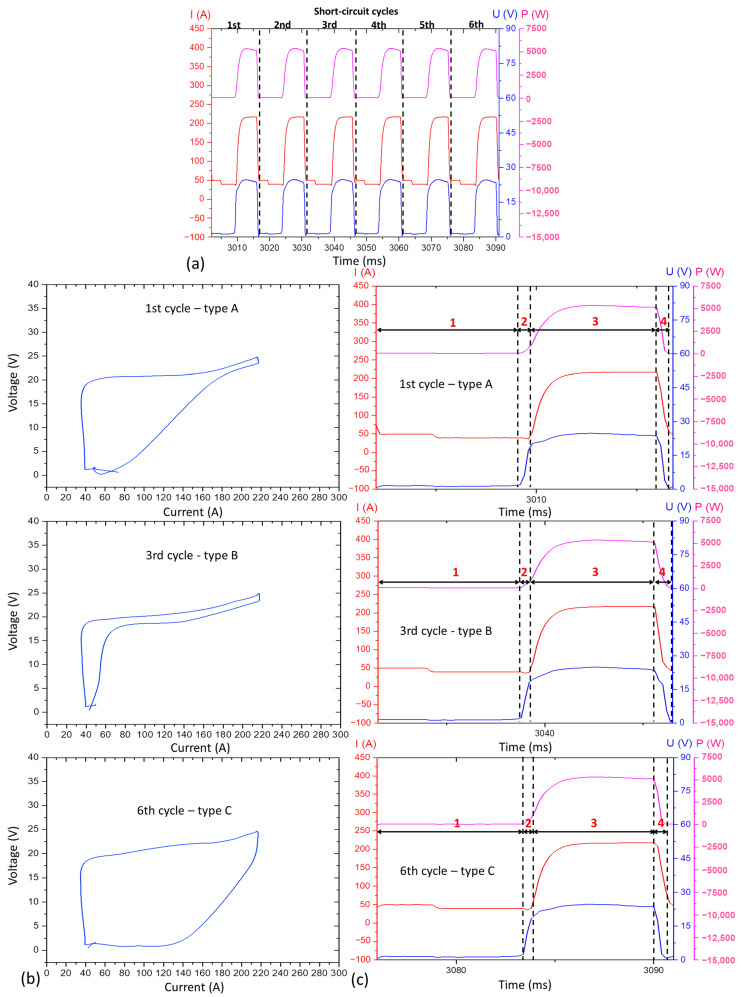
(**a**) Oscillograms of current (I), voltage (U), and power (P) sampling six CMT cycles; (**b**) Three voltage × current cyclograms out of the 1st, 3rd, and 6th cycles, respectively; and (**c**) the oscillograms corresponding to each type of cyclogram identified (A, B, and C), where 1 to 4 are feature regions identified in Figure 4.

Once each region of the cyclogram was identified as in Figure 4, three other cycles were taken from another sampling from the same CMT GMA deposition, as illustrated in Figure 5a. To facilitate the reasoning, this oscillogram image was sliced, and each cycle was sequentially numbered. If each of the six oscillogram cycles are represented as U × I cyclograms, different typical patterns are revealed. The main difference is observed concerning region 4 outlined in Figure 4b. The patterns were categorised based on the trace characteristics of regions 3 and 4, as follows:

- The type A cyclogram occurs when the current and voltage decrease uniformly (constant slop);
- The type B cyclogram shows an abrupt drop in current at high voltage intensity, followed by an abrupt drop in voltage at low current level;
- The type C cyclogram shows an opposite behaviour in relation to type B: an abrupt drop in voltage at high current, followed by an abrupt reduction in current at low voltage.

Figure 5b, the three cyclograms on the left side of Figure 5, shows the three most characteristic cyclogram traces described above. Type A was identified in the 1st, 2nd, and 4th cycles, type B was found during the 3rd and 5th cycles’ analysis, and type C appeared only in the 6th cycle. The more variation of the patterns (types A, B, and C) in the same signal, the higher the trace dispersion, as seen in Figure 3b.

Based on all the reasoning presented, it can be said that Zone θ comes from voltage peaks established at the beginning of arcing time in different cycles, whilst Zone ϕ occurred due to variations in the synchronism between the current and voltage signals within a short time (in Figure 5c, for example, the average of this range was equal to 1.9 ms) that precedes the start of a new short-circuit. Although there were variations in the synchronism of signals, there was the maintenance of arcing and short-circuiting times along with the cycle (Figure 3a), which justifies the low IV_sc_ (higher metal transfer regularity). Thus, any variation regarding the mentioned synchronism (even of a few milliseconds) can result in a significant dispersion in the cyclograms. This finding indicates that the analysis of metal transfer regularity cannot be evaluated only qualitatively using the cyclograms (there is a possibility of misinterpretation). Regardless, both quantitative (IV_sc_ index) and qualitative (oscillograms and cyclograms visual analysis) evaluations continued to be used in this current work to define the set parameters for depositing the walls with Blends 2 and 3, in such a way that a fair comparison of the additives’ effects could fulfil during the “main experiments”.

With that established, similar to those of the reference Blend 1 (Ar + 2%CO_2_), arc length (AL) and dynamic correction (DC) parameters of the CMT process were trimmed to reach the lowest possible spatter formation and a stable metal transfer when using Blend 2 (Ar + 2%H_2_ + 20%He + 500 ppm CO_2_) and Blend 3 (Ar + 1%CO_2_ + 1%H_2_). Table 1 presents the set parameters for comparatively depositing the walls in the “main experiments” stage. Table 1 also presents the monitored and calculated parameters (in columns 5 to 9) averaged after the deposit of 25 layers from each of the three walls. As seen, WFSset had to be higher for Blends 2 and 3 to achieve an I_m_ close to that obtained with Blend 1. Although WFS_m_ was very close among the three walls, the DS settings were slightly adjusted for Blends 2 and 3, because there were trends of variation in WFS_m_ (for less or more depending on the mixture) during the deposition of the first five layers (consequently, results are presented for only 25 of a total of 30 layers). Even so, it is possible to verify that mean current (I_m_) and the ratio of mean wire-feed speed and deposition speed (WFS_m_/DS) are remarkably similar (statistically the same) when comparing the three wall experiments, which satisfies the comparison condition imposed in the methodology (Section 2.1). Notably, the mean voltage (U_m_) was higher with Blend 2. Consequently, even though this blend demanded the fastest deposition speed (DS) to maintain the WFS_m_/DS ratio, a higher energy per unit length was achieved. This behaviour can be explained by the higher heat exchange rate and the higher ionisation potential achieved by adding 20% He. The small addition of hydrogen mimics the effect produced by Blend 3, but to a lesser extent.

### 3.2. From the Main Experiments

Remember that the main experimental stage aimed at comparing the performance of the three gas blends, using the settings from Table 1 (in columns 2 to 4).

#### 3.2.1. Effect of the Shielding Gas on the Regularity of the Short-Circuiting Transfer

Table 2 presents the outcomes from the effect of the chemical composition of the shielding gas on the metal transfer regularity in sort-circuiting mode (Section 2.3.1). Short-circuiting frequency (F_cc_), metal transfer regularity index (IV_sc_), arcing time (t_arc_), short-circuiting (t_sc_) time, and the t_arc_/t_sc_ ratio resulting from 25 layers of the three walls deposited were quantified. A higher short-circuit frequency (F_cc_) was obtained with Blend 1 (Ar + 2%CO_2_), followed by Blend 3 (Ar + 1%CO_2_ + 1%H_2_) and finally by Blend 2 (Ar + 2%H_2_ + 20% He + 500 ppm CO_2_). As mentioned by Jonsson et al. [23] and Lancaster [42], the higher the percentage of oxygen in liquid Fe for the same temperature, the lower the surface tension. Thus, the greater oxidation potential of Blend 1 may have reduced the force due to surface tension in the metallic droplet adhered to the wire tip, making it less resistant to detachment and, consequently, increasing the F_cc_. The same is valid for Blend 3, but on a smaller scale, since the CO_2_ content is lower. In addition, lower IV_sc_ was obtained with Blends 1 and 3, indicating greater regularity of metal transfer in relation to Blend 2 (although still with a very regular metal transfer by the index). The t_arc_/t_sc_ ratios were the same or very close among the three shielding gases evaluated, since this parameter is governed by the arc equipment synergic line, which was the same for the three shielding gases. As t_arc_/t_sc_ ratios were slightly less than one, more than half of the cycles correspond to short-circuit times. This behaviour can be changed according to the wire material and the synergic line.

**Figure 6:**
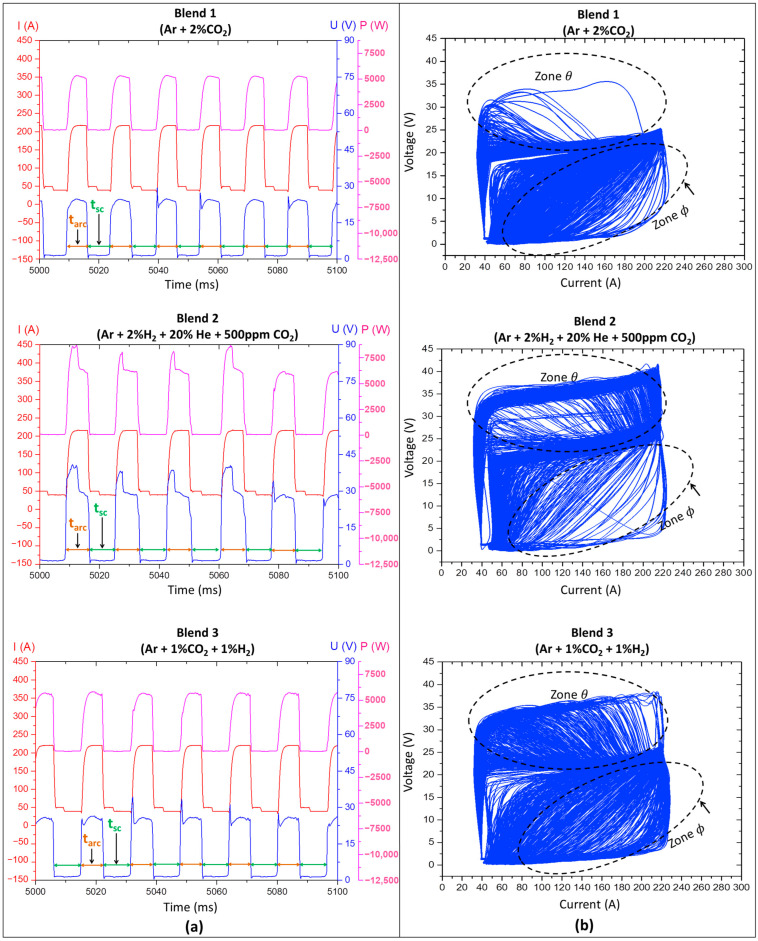
(**a**) Oscillograms of current (I), voltage (U), and power (P), where the brown arrows indicate the arcing-time (t_arc_) semi-period, and the green arrows indicate the short-circuiting time (t_sc_) semi-period; (**b**) cyclograms of the 15th layer sampling.

Considering the significant effect of one of the blends (Blend 2) on the metal transfer regularity, the qualitative analysis of metal transfer was also employed in this assessment. Figure 6a presents the oscillograms sampled (an interval of 100 ms) in the 15th layer of each deposited wall. Evaluating the duration of each half-cycle (highlighted arrows indicating the average t_arc_ (brown arrows) and t_sc_ (green arrows)), the low standard deviation of t_arc_ and t_sc_ of Table 2 is visually confirmed. However, it is difficult to distinguish which shielding gas is more regular by the oscillograms.

A second approach to assess metal transfer regularity by visualising the electrical signals is through cyclograms. Observing Figure 6b, there is high dispersion between the voltage × current curves within the two zones (θ and ϕ) of the cyclograms, regardless of the shielding-gas type. As already mentioned, zone θ results from voltage peaks established at the beginning of arcing time in different cycles. As these peaks were more frequent and longer with Blends 2 and 3 (see Figure 6a), there is a greater dispersion due to these two gases within this zone. This behaviour can be explained by the higher ionisation potential achieved by adding He and H_2_. Zone ϕ, in turn, is established by variations in the synchronism between the current and voltage signals in a short time interval that precedes a new short circuit. In this case, all shielding gases showed great dispersion within zone ϕ. Again, this analysis was not conclusive. The qualitative analysis based on electrical signal visualisation was not helpful, underscoring the importance of using the IV_sc_ index to quantify differences.

#### 3.2.2. Effect of the Shielding Gas on the Geometrical Features

**Figure 7:**
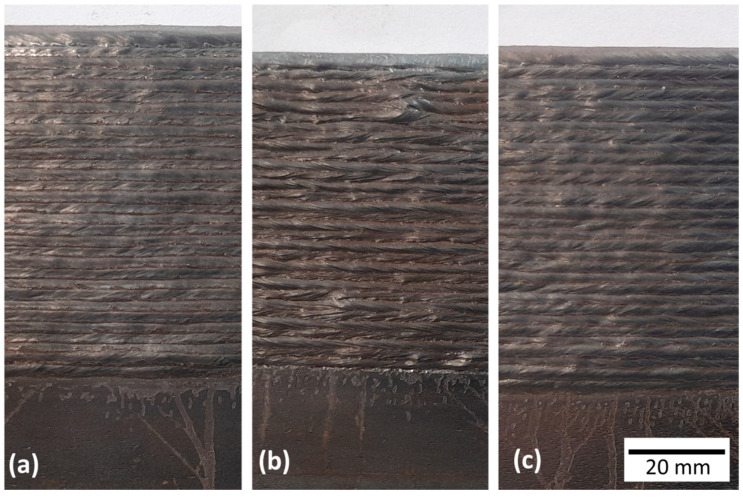
Surface finish (after cleaning with a steel brush) of the walls, using as shielding gases: (**a**) Blend 1 (Ar + 2%CO_2_); (**b**) Blend 2 (Ar + 2%H_2_ + 20%He + 500 ppm CO_2_); and (**c**) Blend 3 (Ar + 1%CO_2_ + 1%H_2_).

Figure 7 shows the surface finish of each wall after cleaning with a manual steel brush. As visually observed, the walls deposited with Blend 1 and Blend 3 had a flatter appearance than those of Blend 2. Higher arc energy (Table 1) due to a gas with higher heat capacity is prone to deliver higher heat input, so lower cooling rates might be obtained with Blend 2 (heat transferred from the arc atmosphere to the pool and surroundings). Consequently, the viscosity of the molten pool is likely smaller and, therefore, less resistant to the movement induced by arc pressure. It is worth mentioning that although the average currents are the same or very close among the different gases (Table 1), the pressure exerted by the plasma jet might be lower with Blend 2 due to the lower density of H_2_ and He (other factors such as variation in the geometry of the electric arc also affect this pressure). Thus, even with less pressure on the pool, the fact that there is a less viscous molten pool was likely responsible for forming a more uneven surface.

**Figure 8:**
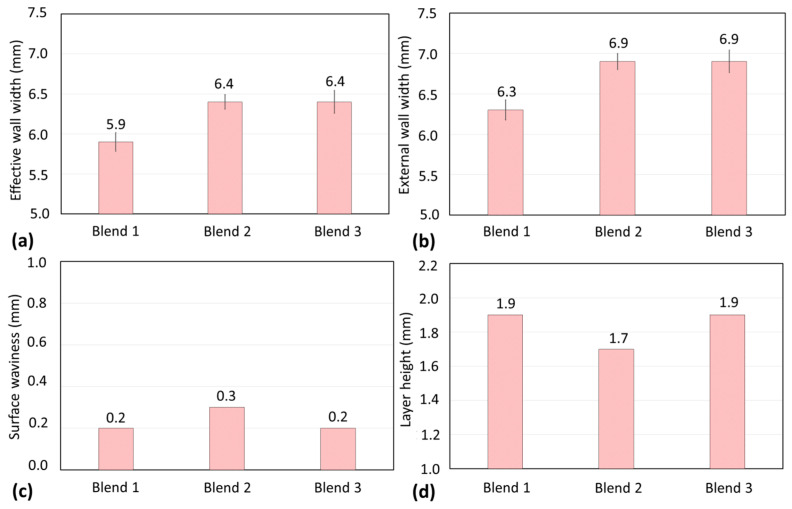
Geometric characteristics of the main experiments: (**a**) effective wall width; (**b**) external wall width; (**c**) surface waviness; and (**d**) layer height (standard deviations for surface waviness and layer height were smaller than the resolution of the measurement instruments detailed in Section 2.3.3).

Figure 8 presents the quantified geometric characteristics represented by the effective wall width (WW_eff_), external wall width (WW_ext_), surface waviness (SW), and layer height (LH). As discussed above, Blend 2 (Ar + 2%H_2_ + 20%He + 500 ppm CO_2_) was prone to lower cooling rates. Consequently, greater effective and external widths in the wall were expected to be deposited with Blend 2, but with lower layer height in relation to the other shielding gases. However, such behaviour was only observed when compared to Blend 1 (Ar + 2%CO_2_). As seen in Table 1, the WFS_m_/DS ratio was a little higher on average for Blend 3 (Ar + 1%CO_2_ + 1%H_2_), leading to a greater amount of deposited material and an increase in those dimensions (probably for the width, since the LH coincides with that of Blend 1, satisfying the proposed explanation). Surface waviness, in turn, was the same when Blends 1 and 3 were used, but a little worse with Blend 2. This result agrees with the appearance of the walls’ surface, shown in Figure 7.

#### 3.2.3. Effect of the Shielding Gas on Total Deposition Time

**Figure 9:**
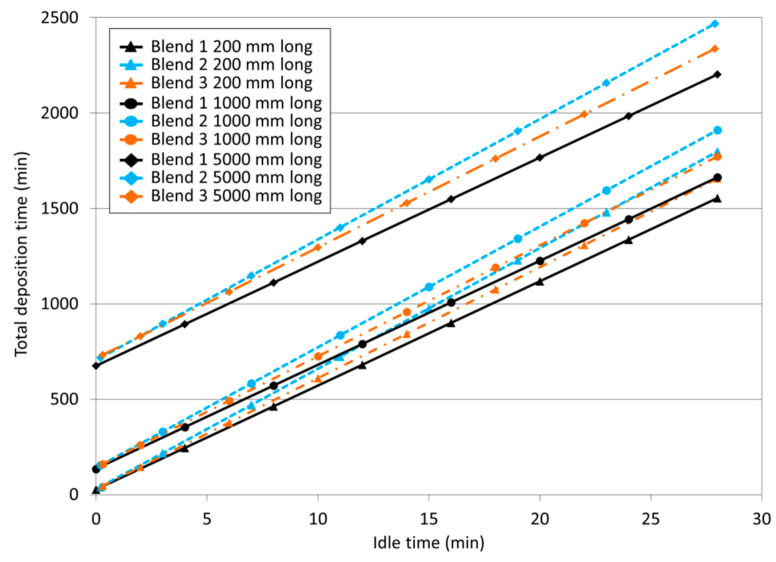
Total deposition times (T_Dt_) for different layer lengths (triangular, circular, and diamond markers consider lengths of 200, 1000, and 5000 mm, respectively) using Blend 1 (Ar + 2%CO_2_), represented by black continuous lines, Blend 2 (Ar + 2%H_2_ + 20%He + 500 ppm CO_2_) by blue dotted lines, and Blend 3 (Ar + 1%CO_2_ + 1%H_2_) by orange dashed and dotted lines.

Figure 9 presents the results of the total deposition time (T_Dt_), considering different idle times (I_t_), hypothetically defined, and wall lengths. Naturally, the longer the I_t_ and wall length, the longer the T_Dt_, regardless of the shielding gas. T_Dt_ was always shorter for the same layer length with Blend 1 (black lines), followed by Blend 3 (orange lines). In this way, Blend 2 delays the production of a wall with the same dimensions (length, height, and width). In addition, the longer the I_t_, the more significant the difference among the evaluated gases, since the lines are further apart. These outcomes emphasise the importance of using forced cooling to speed up production (not used here, but applicable). They also show the effect that different gases can have on T_dt_.

#### 3.2.4. Effect of the Shielding Gas on the Microstructure and Hardness

**Figure 10:**
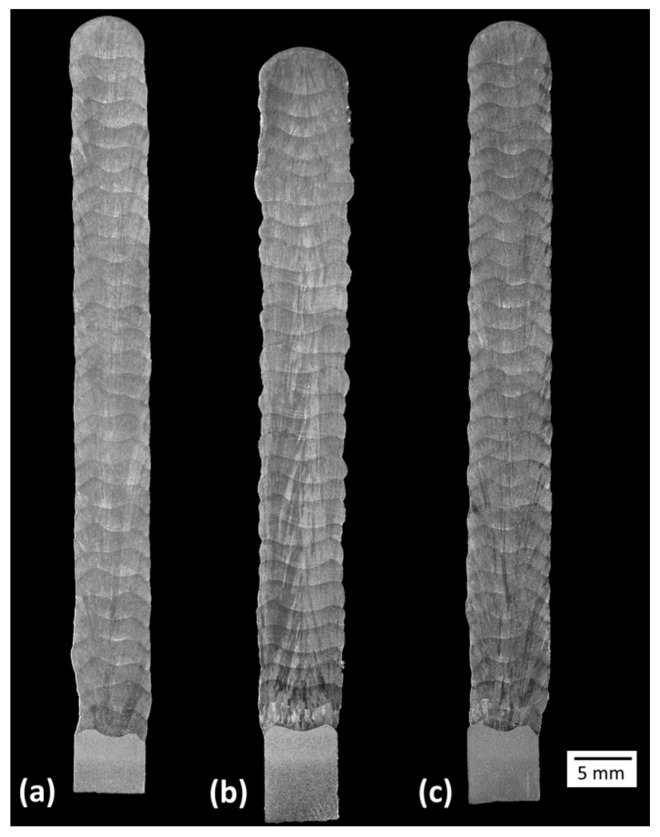
Cross-section macrograph of the walls: (**a**) Blend 1 (Ar + 2%CO_2_); (**b**) Blend 2 (Ar + 2%H_2_ + 20%He + 500 ppm CO_2_); and (**c**) Blend 3 (Ar + 1%CO_2_ + 1%H_2_).

**Figure 11:**
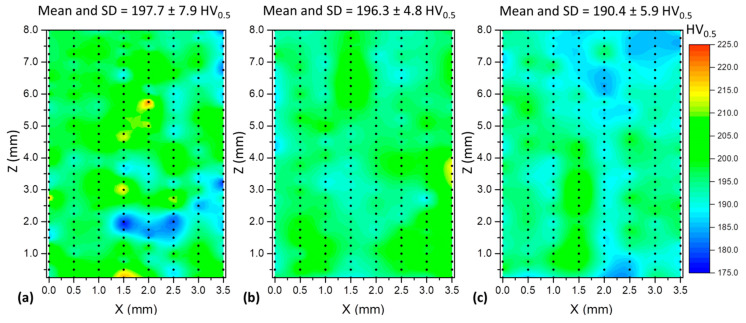
Microhardness maps of the walls: (**a**) Blend 1 (Ar + 2%CO_2_); (**b**) Blend 2 (Ar + 2%H_2_ + 20%He + 500 ppm CO_2_); and (**c**) Blend 3 (Ar + 1%CO_2_ + 1%H_2_) (the black dots represent the indentation positions).

Figure 10 presents macrographs of the three experimental thin walls. No discontinuities (lack of fusion, cracks, porosities) were observed under optical microscopy. These images also emphasise the smoother lateral surface finish (related to waviness) obtained with Blends 1 (Ar + 2%CO_2_) and 3 (Ar + 1%CO_2_ + 1%H_2_), when compared with that obtained with Blend 2 (Ar + 2%H_2_ + 20%He + 500 ppm CO_2_), confirming what was stated previously from Figure 7. Figure 11 shows the microhardness maps, sampled as pointed out in Figure 2. Hardness intensities and distributions are the same or very close to each other (the means and standard deviations are shown at the top of each map). In general, most indentations were concentrated between 190 (light blue) and 205 HV_0.5_ (green), coinciding with the range between 180 and 200 HV_0.5_ found by Wang et al. [43] and also with the result of 205 ± 11 HV_0.2_ found by Feenstra et al. [44], both using DED and the same feedstock as this current work (316L stainless steel).

**Figure 12:**
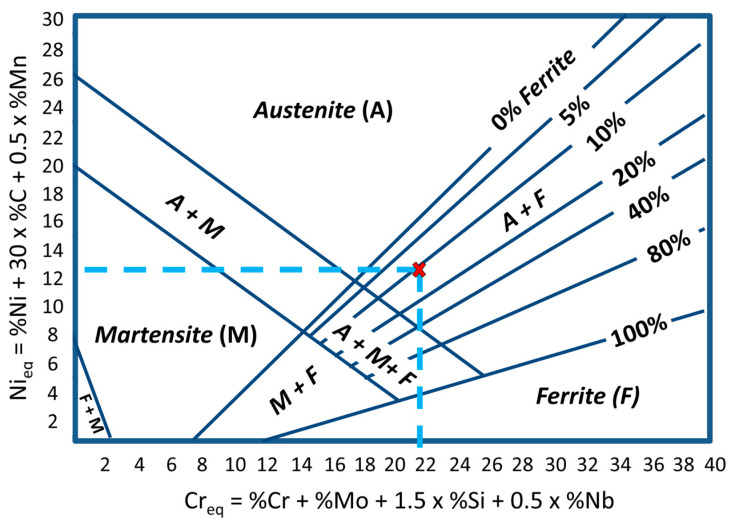
Schaeffler’s constitutional diagram showing the position of the intercept between Cr_eq_ and Ni_eq_ of the wire composition (Table 3).

To provide a better discussion regarding the microstructure results, Table 3 presents the chemical composition of the wire AWS ER316LSi, an austenitic stainless steel. As described by Kou [45], these steels have an austenitic matrix (CFC structure) with variable amounts of δ-ferrite (CCC structure). An adequate amount of δ-ferrite is essential in these materials. A higher content of δ-ferrite (≥10%) tends to reduce ductility, toughness, and corrosion resistance, while δ-ferrite ≤5% makes the microstructure susceptible to solidification cracks. Considering the importance of this phase, the Schaeffler diagram [46] was used to predict the δ-ferrite content in the three deposited walls. The equivalent chromium (Cr_eq_) and nickel (Ni_eq_) were determined based on the chemical composition shown in Table 3. The resultant Cr_eq_ was equal to 21.8%, and Ni_eq_ was equal to 12.7%. As seen in the constitutional diagram of Figure 12, the predicted δ-ferrite content is around 9% (within the ideal range).

**Figure 13:**
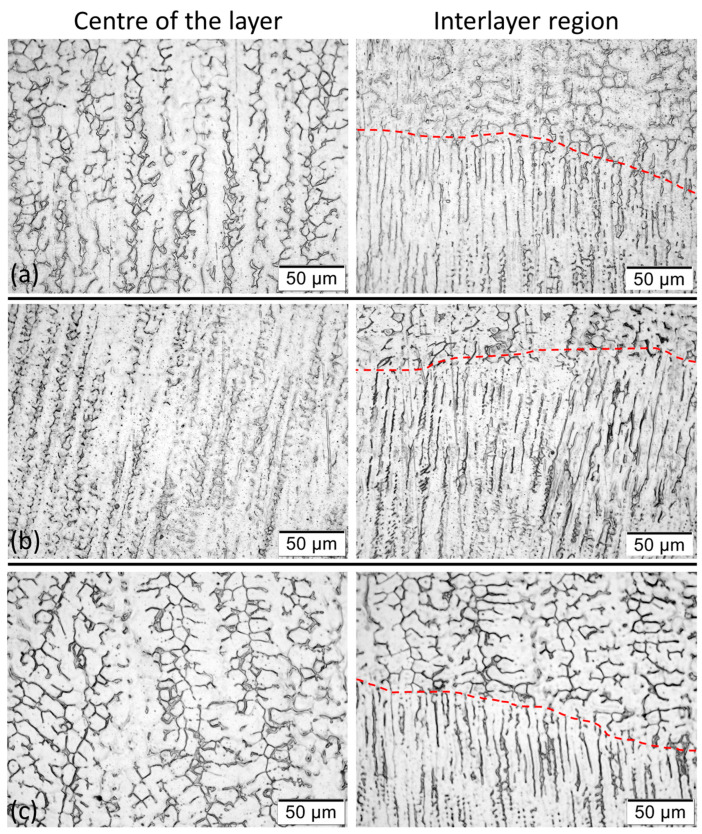
Microstructures (500× magnification and etched with aqua regia) of the walls, using as shielding gas: (**a**) Blend 1 (Ar + 2%CO_2_); (**b**) Blend 2 (Ar + 2%H_2_ + 20%He + 500 ppm CO_2_); (**c**) Blend 3 (Ar + 1%CO_2_ + 1%H_2_) (see positions of them in Figure 2).

Figure 13 illustrates the representative microstructures of the centre (CL) and from the interlayer (IL) region (see referred positions in Figure 2). Considering characterisations already conducted in the literature [35,47,48,49], the dendrites are composed of δ-ferrite (dark phase) and the interdendritic regions by γ-austenite (white phase). This microstructure is typical of FA solidification mode (when solidification occurs as primary ferrite followed by the formation of austenite at the end of solidification). A majority formation of δ-ferrite with vermicular morphology was observed in both evaluated regions, regardless of the shielding gas considered. Corroborating this identification, some microstructures of the FA solidification mode characterised as vermicular ferrite can be seen in Lippold and Kotecki [35] and David [48]. Instabilities in δ-ferrite morphology (transition highlighted by a red line in Figure 13) are also observed, mainly when using Blend 2 and in the interface between layers. Extensive degradation in the micromorphology of the ferrite, particularly the formation of these rugged cylindrical forms, was also observed by David [48] after heat-treating (10 min at 1050 °C) austenitic stainless-steel welds (AWS ER308), which had δ-ferrite vermicular morphology before being treated. As discussed by the author, the formation of such a microstructure may be related to the dissolution of δ-ferrite.

**Figure 14:**
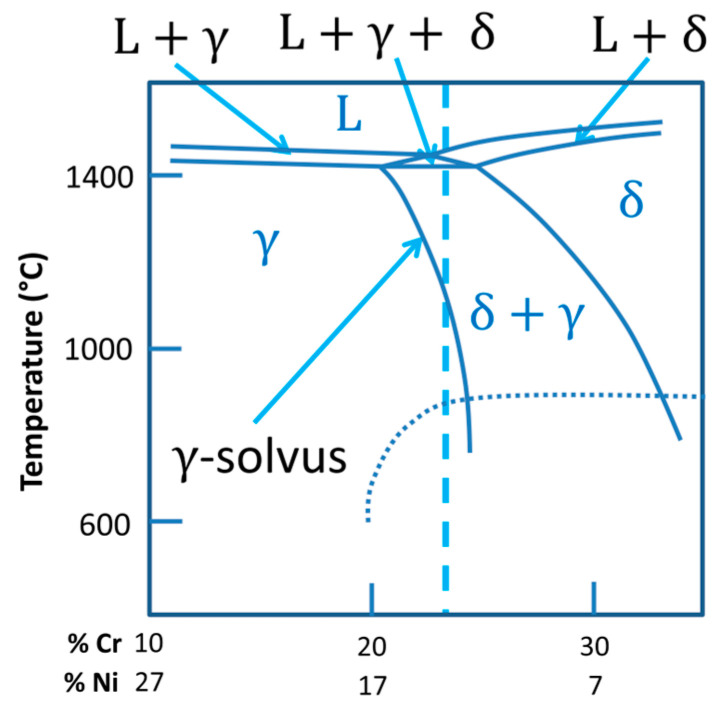
Fe–Cr–Ni pseudo-binary diagram, considering 63% (by weight) Fe and Cr_eq_ = 22% and Ni_eq_ = 13%.

To facilitate the understanding of the micro structuration, Figure 14 presents a Fe–Cr–Ni pseudo-binary diagram considering 63% (by weight) Fe, which was the typical diagram found in the current literature [50], with the closest Fe content to that estimated for the wire used in this work (65.6%). The red dashed line in the diagram encompasses the Cr_eq_ and Ni_eq_ values calculated from Table 3. As seen in Figure 14, only austenite (γ) would be present below the γ-solvus line (indicated by the red arrow). Considering a region with a microstructure as-built, in which δ-ferrite and austenite (γ) co-exist, δ-ferrite may dissolve or suffer degradation when the material is reheated below the γ-solvus line, but it takes time. As several thermal cycles are established during the deposition of the thin wall by GMA, the same region of a given layer can experience varied temperatures below the γ-solvus line, and δ-ferrite can change its morphology or even downsize in these regions. Some publications [51,52] have already described the dissolution of this δ-ferrite present in non-equilibrium conditions, such as in multipass welding or during the repair of austenitic stainless-steel welds.

**Figure 15:**
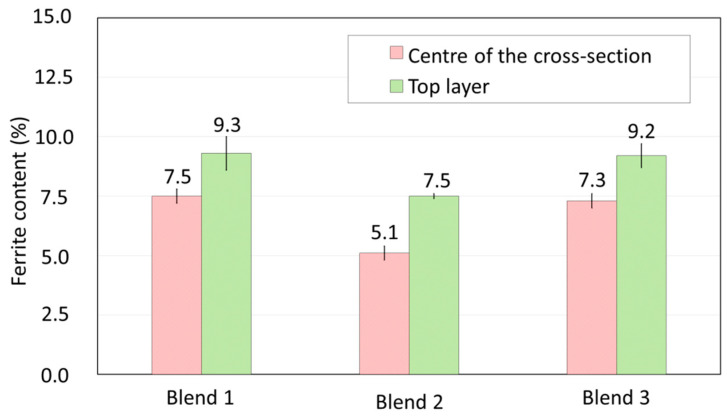
Ferrite content measured using a Feritscope along the centre of the cross-sections and in the last deposited layer (see referred positions in Figure 2).

To complement the discussions, Figure 15 presents the δ-ferrite contents quantified using a Feritscope along the centre length of the walls and in the last deposited layer (see Section 2.3). As seen, the ferrite contents in the last layer were close to the value estimated by the Schaeffler diagram (9%) for Blends 1 and 3. However, there was a reduction of δ-ferrite when Blend 2 was employed to shield the arc. Comparing the measurements of the top deposited layer with the ones in the wall centre, the δ-ferrite content is always lesser in the centre of the wall, indicating a partial dissolution of ferrite in the microstructures of all cross-sections built with the three shielding gases evaluated. It is worth noting that Blend 2 (Ar + 2%H_2_ + 20%He + 500 ppm CO_2_) showed the most significant difference in mean values (2.4%), suggesting consistent greater dissolution of δ-ferrite. Due to the presence of gases with high heat exchange capacity (mainly He at 20% by volume) and also to the higher arc energy during depositions (Table 1), higher heat input may have been obtained with Blend 2. If this occurs, lower cooling rates would be obtained, and longer times below the γ-solvus line could be achieved, even in the top layer, but in particular after multiple thermal cycles. This potential higher heat input would indirectly justify the higher observed ferrite dissolution. 

It should be noted that although chemical composition analyses were not conducted, the possibility of changes in the composition of the walls can also occur. There are potentially two reasons for the variation in composition: CO_2_ in the shielding gas can introduce C to the deposited material, and a higher heat input can lead to greater evaporation of chemical elements. Considering the results found in the literature for the maximum CO_2_ content used in this work (2%CO_2_), regardless of the blend, the change of C pickup by the pool would be minimal. For example, using duplex stainless steel, Zappa et al. [53] identified an increase of 0.001 of C when the Ar + 2%CO_2_ blend was used in relation to an inert blend (Ar + 5%He). In another work, but this time using martensitic stainless steel, Ferreira Filho et al. [9] observed an increase of 0.011 C when the Ar + 2%CO_2_ blend was used as a shielding gas in relation to pure Ar. Furthermore, if there was an increase in the C content, the Ni_eq_ would increase, and a smaller amount of δ-ferrite would be measured. However, shielding with Blend 1 (Ar + 2%CO_2_), with the highest CO_2_ content, presented a higher amount of δ-ferrite than with Blend 2 (Ar + 2%H_2_ + 20%He + 500 ppm CO_2_) and the same as with Blend 3 (Ar + 1%CO_2_ + 1%H_2_). Regarding the second possibility of changing the chemical composition (more evaporation of ferrite-forming elements), there are indications of higher heat input when using Blend 2 (and its 20% He and slightly higher average arc energy), since a smaller amount of ferrite was identified also in the last deposited layer, which reinforces the hypothesis of more significant heat input for this blend.

## 4. Conclusions

The global objective of this work was to propose and evaluate a systematic methodology for shielding-gas selection, using a stainless-steel feedstock as a case study. However, there was a secondary goal, which was to assess and justify the impact of gas components (with different physicochemical properties) used in Ar-based blends recommended as shielding gas for GMA of austenitic stainless steels on the resultant features of the wall when the gases under study were compared. Given both objectives and based on the boundary contour of the experiments (in particular, the feedstock material, the process (CMT version of the GMA) and its synergic line for short-circuiting transfer, and the level of current and arc energy), it was concluded that:

- The proposed methodology, based on a novel, systematic, and comprehensive quantification of the shielding-gas performance (arc- and wall-related characteristics, such as the metal transfer regularity, deposition time, and geometrical and metallurgical aspects of thin walls deposited by GMA), was shown to reach the global objective, by identifying the influence of different commercial shielding gases on various features of GMA (operational, for the process and parametrisation, and functional, concerning the quality and metallurgy of the printed part). Following this methodology, the decision on the shielding selection can be based on the distinguished arc- and wall-related characteristics of the different shielding gases;
- The composition of the gases affects the operational performance of the shielding gases (consequently, metal transfer and fusion rate are governed by the shielding). For a similar deposition rate per unit of layer length and mean current:
  ○ Blend 1 (Ar + 2%CO_2_) and Blend 3 (Ar + 1%CO_2_ + 1%H_2_) provided higher metal transfer regularity than Blend 2 (Ar + 2%H_2_ + 20%He + 500 ppm CO_2_);
  ○ Blend 1 (Ar + 2%CO_2_) leads to shorter total deposition time ($T_{Dt}$), assuming the same idle time between layers, more significantly when the wall length is longer, followed by Blend 3 (Ar + 1%CO_2_ + 1%H_2_) and Blend 2 (Ar + 2%H_2_ + 20%He + 500 ppm CO_2_) in a row (but idle time can be managed and shortened by the shielding gas itself (leading to lower heat input) or externally (active cooling)).
- The composition of the gases affects the heat generated in the arc and that which is transferred to the previous layer (consequently, the layer dimension, wall finish, and metallurgical features are governed by the shielding). For a similar deposition rate per unit of layer length and mean current:
  ○ Blend 1 (Ar + 2%CO_2_) leads to narrower total and effective widths and, consequently, a taller layer height than Blend 2 (Ar + 2%H_2_ + 20%He + 500 ppm CO_2_) and Blend 3 (Ar + 1%CO_2_ + 1%H_2_);
  ○ Blend 1 (Ar + 2%CO_2_) and Blend 3 (Ar + 1%CO_2_ + 1%H_2_) provide similar surface waviness, which was slightly worse when Blend 2 (Ar + 2%H_2_ + 20%He + 500 ppm CO_2_) was shielding the deposition;
  ○ Blend 2 (Ar + 2%H_2_ + 20%He + 500 ppm CO_2_) produces layers with a lower amount of delta ferrite than the other blends. There is no considerable influence of the shielding gas on the microhardness of the build.

For this boundary contour condition, all three assessed shielding gases would be suitable for building thin walls using GMA. However, if the methodology is applied, the blend that generally performed best was Blend 1 (Ar + 2%CO_2_). On the other hand, to emphasise the scientific contribution of this work, it is worth remembering that there are particularities between welding and additive manufacturing (AM), so not every shielding gas successfully used in welding is suitable for AM. This justifies the importance of selecting the most viable gases for AM applications according to the filler metal. However, since evaluating the effect of its change in composition is not trivial, a methodology to facilitate comparison between different gases for WAAM is welcome.

## Figures and Tables

**Table 1:** Set parameters (in columns 2 to 4) used for different shielding gases for comparison purposes (targeting I_m_ = 113 A and WFS_m_/DS ratio = 10.9) and the resulting monitored and calculated variables (in columns 5 to 9).

Gas	WFS_set_ (m/min)	DS (cm/min)	AL	DC	I_m_ (A)	U_m_ (V)	WFS_m_ (m/min)	E_m_ (J/mm)	WFS_m_/ DS
Blend 1	4.9	35.0	−30	−4	112.7 ± 0.8	11.6 ± 0.2	3.8 ± 0.1	367.5 ± 3.7	10.9 ± 0.2
Blend 2	5.7	36.8	−30	−5	110.9 ± 1.4	15.0 ± 0.3	4.0 ± 0.1	442.5 ± 8.7	10.9 ± 0.3
Blend 3	5.2	33.4	−30	−4	111.8 ± 1.1	12.1 ± 0.2	3.8 ± 0.1	401.7 ± 3.6	11.2 ± 0.2

Note: AL = arc length trimming; DC = dynamic correction; WFS = wire-feed speed (set and mean); DS = deposition speed; I_m_ = mean current; U_m_ = mean voltage; E_m_ = averaged instantaneous arc energy.

**Table 2:** Determined metal transfer regularity parameters in short-circuiting mode averaged from 25 layers of each wall.

Gas	WFS_m_ (m/min)	DS (cm/min)	WFS_m_/ DS	F_cc_ (Hz)	IV_sc_	t_arc_ (ms)	t_sc_ (ms)	t_arc_/t_sc_
Blend 1	3.8 ± 0.1	35.0	10.9 ± 0.2	66.9 ± 0.7	0.05 ± 0.02	7.1 ± 0.1	7.8 ± 0.1	0.9 ± 0.0
Blend 2	4.0 ± 0.1	36.8	10.9 ± 0.3	57.8 ± 1.1	0.11 ± 0.03	8.3 ± 0.2	9.0 ± 0.2	0.9 ± 0.0
Blend 3	3.8 ± 0.1	33.4	11.2 ± 0.2	60.8 ± 0.7	0.06 ± 0.02	7.5 ± 0.1	9.0 ± 0.2	0.8 ± 0.0

Note: Mean and standard deviation of short-circuit frequency (F_cc_), metal transfer regularity index (IVsc), arcing time (t_arc_), and short-circuiting time (t_sc_).

**Table 3:** Chemical composition (% in weight) of AWS ER316LSi wire, measured by optical emission spectroscopy (provided by the wire supplier).

C	Mn	Si	S	P	Ni	Cr	Mo	Cu	Fe
0.018	1.770	0.880	0.006	0.024	11.230	18.380	2.080	0.100	Bal.

## Data Availability

The original contributions presented in the study are included in the article; further inquiries can be directed to the corresponding author.
